# Adherence to Updated Race and Ethnicity Reporting Guidance in Ophthalmology Journals

**DOI:** 10.1001/jamanetworkopen.2025.29778

**Published:** 2025-09-02

**Authors:** Vivian Rajeswaren, Bridget Croniger, Karim Dirani, M. Roy Wilson

**Affiliations:** 1 Department of Ophthalmology, Visual and Anatomical Sciences, Wayne State University School of Medicine, Detroit, Michigan; 2 Kresge Eye Institute, Detroit, Michigan

## Abstract

**Importance:**

The updated race and ethnicity reporting guidelines published in the *AMA Manual of Style: A Guide for Authors and Editors* highlight the importance of using specific racial and ethnic categories rather than generalized terms, promoting inclusive language, supporting diversity and respect, and examining sociodemographic factors that shape the relationship between race and ethnicity and health outcomes.

**Objective:**

To evaluate adherence to the *AMA Manual of Style’s* updated race and ethnicity reporting guidelines in the 3 highest-impact ophthalmology journals.

**Evidence Review:**

A systematic review of articles published in *American Journal of Ophthalmology, JAMA Ophthalmology, and Ophthalmology* from August 1, 2023, through August 31, 2024. Articles with a study population were included. Articles were excluded if they lacked a study population, used artificial intelligence, determined race and ethnicity by facial features, or reported ancestry instead of race or ethnicity.

**Findings:**

This review yielded 931 research articles, with 525 meeting inclusion criteria. Of these, 491 articles (93.5%) reported age, 472 (89.9%) sex and/or gender, 285 (54.3%) race and/or ethnicity, and 96 (18.3%) socioeconomic measures. Of the 285 articles reporting race and/or ethnicity, 159 (55.8%) reported “race,” 155 (54.4%) “ethnicity,” and 106 (37.2%) “race/ethnicity” as 1 category. Race was misclassified as ethnicity or vice versa in 54 articles (19.0%); 3 (5.6%) used primary datasets, 13 (24.1%) used secondary, and 38 (70.4%) did not specify. A total of 276 articles (96.8%) reported specific race and/or ethnicity categories; 19 articles (6.9%) collected race and ethnicity data without reporting categories. Race and ethnicity categories were used as nouns in 44 articles (15.4%). “Minority/minorities” was used in 39 articles (13.7%), and the use of a modifier was mixed, with a modifier consistently used in 22 (56.4%), inconsistently in 4 (10.3%), and not at all in 13 (33.3%). Of the 26 articles (9.1%) using “multiracial” or “multiethnic,” 5 (19.2%) reported the included categories. “African American” and “Black” were used interchangeably in 23 articles (8.1%), and 36 (12.6%) used “Caucasian” for White race.

**Conclusions and Relevance:**

In this systematic review of research articles in the 3 highest-impact ophthalmology journals from August 2023 through August 2024, race and ethnicity reporting was substantially nonadherent with updated *JAMA* recommendations. These findings suggest that improvements in demographic reporting are needed.

## Key Points

**Question:**

How adherent are the 3 highest-impact ophthalmology journals with the *AMA Manual of Style’s* updated guidance on the reporting of race and ethnicity?

**Findings:**

This systematic review of 525 research articles published from August 1, 2023, to August 31, 2024, in *American Journal of Ophthalmology, JAMA Ophthalmology,* and *Ophthalmology* demonstrated substantial nonadherence with *JAMA’s* updated guidance.

**Meaning:**

These findings suggest that there is an opportunity for ophthalmology journals to improve current race and ethnicity reporting practices to better identify and address health disparities.

## Teaser

This systematic review evaluates adherence to the updated race and ethnicity reporting guidelines from the *AMA Manual of Style* in 3 high-impact ophthalmology journals from 2023 to 2024.

## Introduction

The role of race and ethnicity in medical research is controversial, with ongoing debate regarding the biological validity and appropriate use of these categories.^1^ It is widely acknowledged that race is a sociopolitical construct with limited biologic importance.^2,3^ However, substantial disparities in health care outcomes based on race and ethnicity are well documented, and it is important to report racial and ethnic information to identify and reduce these inequities.^4,5,6,7,8^ Therefore, organizations such as the National Institutes of Health and the International Committee of Medical Journal Editors advise authors to include a representative study population and describe the racial and ethnic composition of participants, while also explaining the relevance of these categories to their research.^2,9^

There are concerns regarding appropriate methods for the inclusion of race and ethnicity in biomedical investigations.^10^ Bonham and colleagues^11^ caution that imprecise use of race and ethnicity data can misrepresent the complexities among health, social identity, ancestry, and socioeconomic status and reinforce the misconception that race corresponds to distinct biologic groups. Classifying patients using oversimplified racial and ethnic categories can lead to harmful assumptions, such as race-based interpretations of pulmonary and kidney function, which have contributed to disparities in health care.^12,13^

Historically, race was used as a broad category to divide individuals based on ancestry and physical characteristics, whereas ethnicity refers to cultural identity.^3,14^ Genomic research has demonstrated that physical traits used to define racial groups do not align with biological differences and ongoing population mixing further undermines any meaningful genetic distinctions.^12^ It is now largely accepted that definitions based on physical characteristics are outdated and may lead to systemic discrimination.^3^

To address these inequalities and improve patient outcomes, the scientific community has advocated for standardized recommendations for collecting and reporting patient demographics.^10^ However, previous studies^15,16,17,18,19,20^ have demonstrated low rates of race and ethnicity reporting and limited adherence to published recommendations across medical specialties.

In 2021, a report in *JAMA* presented updated recommendations from the *AMA Manual of Style* for standardized, precise, and equitable reporting of race and ethnicity.^3^ These guidelines emphasize the importance of using specific racial and ethnic categories rather than broad or generalized terms, promoting inclusive language that supports diversity and respect, and examining sociodemographic factors that influence the relationship between race and ethnicity and health outcomes.^3^ Flanigan and colleagues^21^ compared race and ethnicity reporting in *JAMA*, *JAMA Internal Medicine*, and *JAMA Pediatrics* before and after implementation of this updated guidance and found an increase in the proportion of articles reporting how race and ethnicity were determined and how racial and ethnic categories were defined but no significant improvement in other areas. These findings highlight that although progress in race and ethnicity reporting has been achieved, further improvement is needed.

On October 30, 2024, a consensus committee of the National Academies of Sciences, Engineering, and Medicine (NASEM), chaired by the senior author (M.R.W.), released a study report on the use and misuse of race in biomedical research.^22^ Titled *Rethinking Race and Ethnicity in Biomedical Research*, the report reinforces many of the updated race and ethnicity reporting guidelines issued by *JAMA*.^22^ Furthermore, the NASEM report advocated for consistent reporting guidelines across journals and disciplines and called for more accountability and enforcement of guidelines throughout the publication process.^22^ Our study evaluates adherence to *JAMA*’s updated race and ethnicity reporting guidelines in 3 high-impact ophthalmology journals: *American Journal of Ophthalmology (AJO)*, *JAMA Ophthalmology,* and *Ophthalmology,* from August 2023 to August 2024.

## Methods

This systematic review was exempt from informed consent and ethical review in accordance with US regulations (45 CFR §46) for studies without human participants and adhered to the Preferred Reporting Items for Systematic Reviews and Meta-analyses (PRISMA) reporting guideline and the Meta-analysis of Observational Studies in Epidemiology (MOOSE) reporting guideline. All major research articles published in *AJO*, *JAMA Ophthalmology*, and *Ophthalmology* from August 1, 2023, to August 31, 2024, were included (eTable in Supplement 1). Two reviewers (B.C. and V.R) independently assessed and excluded articles without a study population, such as editorials, perspectives, commentaries, case reports, and certain literature reviews. Studies using artificial intelligence to predict outcomes or learn from datasets as well as articles reporting patient ancestry instead of race or ethnicity were excluded. An article using facial morphologic features to identify ethnicity was removed because the reported ethnicity was not comparable to other studies. Additionally, articles that used artificial intelligence–generated datasets were excluded because they lacked a comparable study population.

Articles were independently assessed by 2 reviewers (B.C. and V.R.) for the following variables based on key points from *JAMA’s* updated guidelines: inclusion of human participants; reporting of age, sex, gender, race and/or ethnicity, race and ethnicity as a single category, and measures of socioeconomic status (SES); location of race and ethnicity information (eg, Abstract, Methods section, Results section, tables, and supplement); demographic variables collected but not reported and the reason; whether race and ethnicity classifications were derived specifically for the study or were determined from historical data from past studies; who determined race and ethnicity and where this was noted in the article; source of race and/or ethnicity and location in the article; reason for collecting race and/or ethnicity categories and where this was mentioned in the article; number and order of racial and ethnic categories reported; whether the category “other” was included and if it was defined; whether race and/or ethnicity categories were listed in alphabetical order; whether race and/or ethnicity categories were capitalized; use of race and/or ethnicity categories as adjectives or nouns; use of the term “mixed race” for data collection and whether the specific races were defined; use of the terms “minority” or “minoritized” and whether a modifier such as “racial” or “ethnic” was included; inclusion of the terms “multiracial” or “multiethnic”; whether the specific races or ethnicities were defined and whether these terms were selected from a study database; and whether the terms “Arab” and “Arab American,” “Asian,” and “Asian American” were used interchangeably and whether the terms “African American” and “Black” were used interchangeably without defining both terms. The use of the term “Caucasian” to designate White individuals outside the Caucasus region was also recorded. Variables were determined based on *JAMA’s* updated race and ethnicity reporting guidelines.^3^

Discrepancies in data collection were resolved by 2 additional reviewers (K.D. and M.R.W.) in biweekly meetings as detailed in the following paragraph. SES indicators were defined as income, educational level, housing, health insurance, and employment. The categories “race/ethnicity” and “race and ethnicity” were considered equivalent. Articles reporting race/ethnicity as a combined category were not counted as reporting either race or ethnicity separately. If an article reported racial or ethnic information without specifying whether a category was classified as a race or ethnicity, reviewers determined the classification based on the Office of Management and Budget guidelines.^23,24^ When race or ethnicity data were collected specifically for the study, the outcome was classified as primary. When they were derived from past studies wherein the data were static after study completion, the outcome was classified as secondary. Studies that did not describe the details of data capture (eg, with electronic medical records wherein race and ethnicity are reported without clarifying whether classifications reflected patient self-identification or if predetermined categories were used) were coded as unspecified. When an individual’s country of origin was reported but their ethnicity was not explicitly stated, the outcome was not classified as reporting ethnicity information. If a study indicated that a variable was described in another section of the article, the recorded location was the section containing the listed data. Descriptions of the study design or methods that referenced prior publications were not recorded in our dataset. Race and ethnicity information estimated from population values were considered the same as reporting race and ethnicity of the study cohort. Articles listing race and/or ethnicity as demographic variables were counted as reporting race/ethnicity in that section of the article. The source, method of collection, and rationale for collecting race and ethnicity data were recorded when reported for all demographic variables or specifically for race and ethnicity information. If a rationale was provided for analyzing data by race and ethnicity categories, it was noted as the reason for collecting race and ethnicity variables. Articles categorizing race into underrepresented in medicine (URiM) and non-URiM groups were coded as reporting racial information if definitions of these groups were provided. The alphabetical order of racial and ethnic groups was based on category order, excluding the categories “other” and “unknown.” Alphabetical order within specific categories (eg, American Indian or Alaska Native) was not assessed. Racial descriptors used as nouns and adjectives were coded identically whether referring to the study population or discussing another article. Articles using the combined category “African American or Black” with subsequent use of only “Black” or “African American” were recorded as using the terms interchangeably.

## Results

From August 1, 2023, to August 31, 2024, there were 931 research articles published in *AJO*, *JAMA Ophthalmology*, and *Ophthalmology* and 525 articles met the inclusion criteria (Figure). Table 1 describes reported demographic characteristics and the location of reporting. Of the 525 included articles, 491 (93.5%) reported age, 472 (89.9%) reported sex and/or gender, 285 (54.3%) reported race and/or ethnicity, and 96 (18.3%) reported SES measures. For the 285 articles reporting race and/or ethnicity, the specific racial and ethnic categories, rationale, methods, and reporting location were recorded. For specific categories, 159 (55.8%) reported “race,” 155 (54.4%) reported “ethnicity,” 106 (37.2%) reported “race/ethnicity” as one category, and 54 (19.0%) misclassified race as ethnicity or ethnicity as race; 3 (5.6%) used primary datasets, 13 (24.1%) used secondary datasets, and 38 (70.4%) did not specify. Regarding the location of reporting, 102 articles (35.8%) reported race and/or ethnicity in the Abstract, 174 (61.1%) in the Methods section, 187 (65.6%) in the Results section, and 216 (75.8%) in a table. Data for race and ethnicity classification were categorized as derived from primary (27 [9.5%]), secondary (69 [23.8%]), and unspecified (190 [66.6%]) datasets. Reporting who identified participant race and/or ethnicity occurred in 91 articles (31.9%); 71 (78.0%) reported it in the Methods section and 60 (65.9%) in other sections. Defining the source of race and/or ethnicity was observed in 170 articles (59.7%); 162 (95.3%) reported the source in the Methods section, and 79 (46.5%) in other sections. The reason for collecting race and ethnicity information was reported in 116 articles (40.7%); 68 (58.6%) reported a reason in the Methods section and 77 (66.4%) in other sections. Specific race and/or ethnicity categories were reported in 276 articles (96.8%); 19 (6.9%) of these collected race and ethnicity data but did not report specific categories, and 8 of the 19 (42.1%) specified a reason for not reporting race and ethnicity categories collected. An “other” category was used in 143 articles (50.2%); 40 (28.0%) defined the races or ethnicities included. With respect to alphabetization, 66 articles (23.2%) listed categories alphabetically in the text or in the tables.

**Figure.**
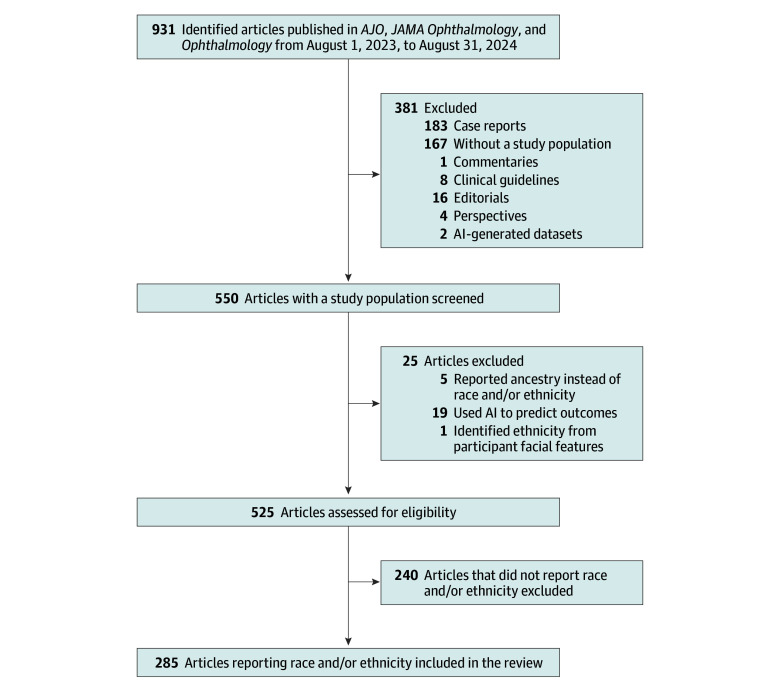
Flow Diagram of the Study Selection Process AI indicates artificial intelligence; *AJO*, *American Journal of Ophthalmology.*

**Table 1.** Demographic Characteristics of the Included Articles

Characteristic	No./total No. (%) of articles
Demographic characteristic reported	
Age	491/525 (93.5)
Sex and/or gender	472/525 (90.0)
Race and/or ethnicity	285/525 (54.3)
Socioeconomic measures	96/525 (18.3)
Race and/or ethnicity terms reported	
Race	159/285 (55.8)
Ethnicity	155/285 (54.4)
Race/ethnicity as a single category	106/285 (37.2)
Misclassified race as ethnicity or ethnicity as race	54/285 (19.0)
Location of race and/or ethnicity reporting	
Abstract	102/285 (35.8)
Methods	174/285 (61.1)
Results	187/285 (65.6)
Table	216/285 (75.8)
Type of dataset	
Primary	27/285 (9.5)
Secondary	69/285 (23.8)
Unspecified	190/285 (66.6)
Indicated who identified race and/or ethnicity	
Total	91/285 (31.9)
Methods section	71/91 (78.0)
Other section	60/91 (66.0)
Indicated source of race and/or ethnicity classifications	
Total	170/285 (59.7)
Methods section	162/170 (95.3)
Other section	79/170 (46.5)
Defined reason for collecting race and/or ethnicity data	
Total	116/285 (40.7)
Methods section	68/116 (58.6)
Other section	77/116 (66.4)
Categories reported	
Reported racial and/or ethnic categories	276/285 (96.8)
Did not collect or report racial and/or ethnic categories	19/276 (6.8)
Specified a reason for not reporting race and/or ethnicity categories collected	8/19 (42.1)
Included an other category	143/285 (50.2)
Defined categories included in the other category	40/143 (28.0)
Presented categories in alphabetical order in text or tables	66/285 (23.2)

Table 2 details reporting characteristics pertaining to the use of racial and ethnic collective terms in the 285 articles reporting race and/or ethnicity. Capitalization of racial and ethnic categories was recorded in 260 articles (91.2%). Race and ethnicity categories were used in noun and adjective form in 44 articles (15.4%) and 160 articles (56.1%), respectively. Use of “minority/minorities” was observed in 39 articles (13.7%); 22 (56.4%) consistently used a modifier, 4 (10.3%) used one inconsistently, and 13 (33.3%) did not use a modifier. The term “mixed race” used for data collection was recorded in 6 articles (2.1%). “Multiracial” or “multiethnic” was noted in 26 articles (9.1%); 5 (19.2%) reported the included categories. “African American” and “Black” were used interchangeably in 23 articles (8.1%), and “Caucasian” was used for White race in 36 articles (12.6%). Table 3 summarizes the 10 most frequent race and ethnicity reporting misuses identified across the 3 journals.

**Table 2.** Reporting Characteristics Pertaining to the Use of Racial and Ethnic Collective Terms in Articles Reporting Race and/or Ethnicity

Characteristic	No./total No. (%) of articles
Capitalized race and/or ethnicity categories	260/285 (91.2)
Used race and/or ethnic categories as nouns	44/285 (15.4)
Used race and/or ethnic categories as adjectives	160/285 (56.1)
Used “African American” and “Black” interchangeably	23/285 (8.1)
Used “Caucasian” to denote White race	36/285 (12.6)
Used “mixed race” for data collection	6/285 (2.1)
Used the “multiracial” or “multiethnic”	26/285 (9.1)
Reported included racial and/or ethnic categories for “multiracial” or “multiethnic”	5/26 (19.2)
Used “minority” and/or “minorities”	39/285 (13.7)
Used a modifier consistently	22/39 (56.4)
Used a modifier inconsistently	4/39 (10.3)
Never used a modifier for the term minorities, such as racial or ethnic minorities	13/39 (33.3)

**Table 3.** Most Frequent Misuses of Race and Ethnicity Reporting in the 3 Highest-Impact Ophthalmology Journals

Characteristic	No. (%) of articles (N = 285)
Did not present categories in alphabetical order in tables and/or text	219 (76.8)
Did not indicate who identified race and/or ethnicity	194 (68.1)
Did not define the reason for collecting race and/or ethnicity categories	169 (59.3)
Used race and/or ethnicity categories as adjectives	160 (56.1)
Included other category	143 (50.2)
Did not define the source of race and/or ethnicity classification	115 (40.3)
Misclassified race as ethnicity or ethnicity as race	54 (19.0)
Used race and/or ethnicity categories as nouns	44 (15.4)
Inconsistently used a modifier for the term *minorities,* such as racial and ethnic minorities	4 (10.3)
Never used a modifier for the term *minorities,* such as racial and ethnic minorities	13 (33.3)
Used *Caucasian* to denote White race	36 (12.6)
Used *African American* and *Black* interchangeably	23 (8.1)

## Discussion

This review of articles published in 3 major ophthalmology journals during a 13-month period found a low frequency of race and ethnicity reporting. More than 90% of articles reported age and sex or gender, but only approximately half reported race and/or ethnicity, a trend seen across medical fields. Moore et al^15^ found that 88% of articles published in 2019 in *AJO, JAMA Ophthalmology,* and *Ophthalmology* reported age and sex, but only 43% included race and ethnicity. Similar results were observed for race and ethnicity reporting in surgery (32.5%) in 2019.^16^ Race and ethnicity are social constructs with limited biological basis, and their indiscriminate use in clinical algorithms reinforces biases and can lead to errors in diagnosis and treatment.^25^ Nonetheless, these variables capture important epidemiologic information, including the impact of racism, socioeconomic disparities, and environmental exposures^3,26^; accurate tracking of race and ethnicity data is thus crucial for identifying health disparities and informing efforts to advance equity in health outcomes.^27,28^

Although ethnicity has historically referred to cultural identity and race to categories based on ancestry and physical traits, definitions relying on physical characteristics are inherently flawed and can perpetuate racism.^3,6^ Additionally, significant overlap in general use of the terms “race” and “ethnicity” complicates the distinction between these terms.^6^ The use of 2 questions for race and ethnicity in data collection, as has been the case with Hispanic individuals, can force the selection of a less specific category or lead to higher rates of nonresponse, compromising clinical outcomes and identification of health inequalities.^29,30,31^ In 2016, the US Census Bureau found that a combined race and ethnicity question increased the number of Hispanic and Latino respondents identifying solely as Hispanic rather than choosing imprecise categories such as “White” or “other.”^31^ Therefore, in accordance with updated *JAMA* guidelines, the use of a combined “race and ethnicity” category that permits multiple selections is recommended.

Additionally, researchers should report specific racial and ethnic categories and define the populations included in any group labeled “other.”^3^ Our study found that 37.2% of articles reported “race/ethnicity” as a single category. Prior research indicated that in 2019 a total of 33.1% of surgery articles published in journals adhering to International Committee of Medical Journal Editors guidelines and 33% of ophthalmology articles in the 3 highest-impact journals reported race and ethnicity as a single category.^15,16^ Additionally, our analysis found that 50.2% of articles included an “other” category for race and ethnicity, with 40 (28.0%) providing definitions for the included categories. Flanigan and colleagues^21^ found that in 2022 a total of 61.1% of internal medicine and pediatric articles classified race and/or ethnicity as “other,” and 84.8% of these defined the categories included.

Accurate reporting of participant race and ethnicity, alongside other sociodemographic data, allows for a more comprehensive understanding of study populations.^3^ Some nonadherence with reporting guidance in our study was attributable to limitations in the secondary dataset used. In these circumstances, investigators are typically bound to use the data as reported at the time of initial data collection. However, to avoid perpetuating misuses in how race and ethnicity are categorized and used, we urge authors to discuss the limitations in these datasets. Moreover, misclassification of race and ethnicity variables directly affects research findings, potentially hindering development of effective interventions and provision of equitable care.^32^ We found that 19.0% of articles misclassified race as ethnicity or vice versa. This value is lower than the previously reported value of 38% of articles misclassifying race as ethnicity or ethnicity as race in the same 3 journals in 2019.^15^

Acknowledging geographic origin and regionalization in racial and ethnic designations is essential because the use of subpopulation-specific categories provides insight into intragroup differences.^33^ In contrast, broad or interchangeable classifications may obscure meaningful distinctions among subgroups, thereby limiting the accuracy and generalizability of findings.^34^ In our analysis, 8.1% of articles used the terms “African American” and “Black” interchangeably without formal definition of both terms. Another 12.6% of articles in our study used the term “Caucasian” as a racial category. Although this term has historically been used to designate White individuals, it has racist origins and should be limited only to characterizing individuals from a specific geographic area, the Caucasus region in Eurasia.^3,35^

The use of inclusive language for race and ethnicity cultivates respect and avoids perpetuating stereotypes that can negatively impact patient-physician communication and lead to disparities in patient care and clinical outcomes.^36,37,38^ Racial and ethnic categories should be listed in alphabetical order instead of order by majority. The term “minorities” should be avoided due to its inherent hierarchical implications unless accompanied by a clarifying modifier, such as “racial and ethnic minorities.”^3^ Adjectival forms are preferred over noun forms when describing racial and ethnic groups in accordance with AMA guidelines for person-first language, which aim to emphasize the individual and avoid stigmatizing language.^3^ Our review found that 15.4% of articles used race and/or ethnicity categories in noun form and 13.7% of articles used the terms “minority” or “minorities.” Among these articles, 56.4% consistently used modifiers, 10.3% used modifiers inconsistently and 33.3% did not use a modifier. Only 66 articles (23.2%) in this review reported race and ethnicity categories alphabetically in the text or in the tables compared with 92.6% of articles in *JAMA*, *JAMA Internal Medicine*, and *JAMA Pediatrics* published in 2022 after the updated recommendations for reporting race and ethnicity were implemented.^21^

Clear explanations of the methods and rationale for race and ethnicity data collection are essential for research transparency and accurate interpretation of outcomes.^39^ Our results demonstrated a 31.9% rate of reporting how race and/or ethnicity were determined. Rates in other studies vary from 1.2% to 74.1%.^15,17,21,40^ Additionally, 40.7% of articles reported the reason for collecting race and/or ethnicity categories compared with rates of 10.8% to 69.5% in the literature.^16,40^

### Limitations

This study has limitations. Challenges exist in assessing adherence to *JAMA*’s reporting guidelines across journals. Although JAMA Network editors can enforce these standards during peer review, authors publishing in other journals may have limited awareness of the guidelines, and editorial oversight may differ by publication. Additionally, our analysis was limited to articles published in 3 journals, which may not accurately represent race and ethnicity reporting practices for all ophthalmology literature. We also did not conduct comparative analyses of race and ethnicity reporting across journals.

## Conclusions

Race and/or ethnicity reporting in the 3 highest-impact ophthalmology journals (August 2023 to August 2024) was infrequent and not fully adherent to updated *JAMA* and NASEM recommendations. Despite increasing awareness of the importance of precise and comprehensive race and ethnicity reporting in medical and scientific literature, improvements in demographic reporting are needed. Efforts should be made to improve adherence to reporting guidelines to effectively identify and address inequities in health care.

## Notes

Supplement 1. **eTable.** All Major Research Articles Published in AJO, JAMA Ophthalmology, and Ophthalmology From August 1, 2023, to August 31, 2024

Supplement 2.
Data Sharing Statement
