# Supplementary material for: Adherence to Updated Race and Ethnicity Reporting Guidance in Ophthalmology Journals
Source: JAMA Netw Open. 2025 Sep 2;8(9):e2529778. doi: 10.1001/jamanetworkopen.2025.29778 (PMC12406061; doi:10.1001/jamanetworkopen.2025.29778)
Supplement: Supplement 1. — eTable. All Major Research Articles Published in AJO, JAMA Ophthalmology, and Ophthalmology From August 1, 2023, to August 31, 2024 [file jamanetwopen-e2529778-s001.pdf]

# Supplemental Online Content

Rajeswaren V, Croniger B, Dirani K, Wilson MR. Adherence to updated race and ethnicity reporting guidance in ophthalmology journals, 2023-2024. *JAMA Netw Open*. 2025;8(9):e2529778.
doi:10.1001/jamanetworkopen.2025.29778

**eTable.** All Major Research Articles Published in *AJO, JAMA Ophthalmology,* and *Ophthalmology* From August 1, 2023, to August 31, 2024

This supplemental material has been provided by the authors to give readers additional information about their work.

© 2025 Rajeswaren V et al. *JAMA Network Open.*

**eTable.** All Major Research Articles Published in *AJO, JAMA Ophthalmology,* and *Ophthalmology* From August 1, 2023, to August 31, 2024

| **Articles from *JAMA* included in the dataset** | **Articles from *AJO* included in the dataset** | **Articles from *Ophthalmology* included in the dataset** |
|---|---|---|
| Complications Occurring Through 5 Years Following Primary Intraocular Lens Implantation for Pediatric Cataract | Change in Cone Structure Over 24 Months in USH2A-Related Retinal Degeneration | Fourteen-Year Outcome of Angle- Closure Prevention with Laser Iridotomy in the Zhongshan Angle- Closure Prevention Study Extended Follow-up of a Randomized Controlled Trial |
| Prognostic Value of Parenteral Nutrition Duration on Risk of Retinopathy of Prematurity | Visual Outcomes Following Plasma Exchange for Optic Neuritis: An International Multicenter Retrospective Analysis of 395 Optic Neuritis Attacks | Factors Linked to Injection Interval Extension in Eyes with Wet Age- Related Macular Degeneration Switched to Brolucizumab |

© 2025 Rajeswaren V et al. *JAMA Network Open.*

| | | |
|---|---|---|
| Social Risk Factor Associations With Presenting Visual Acuity in Patients With Microbial Keratitis | OCT Optic Nerve Head Morphology in Myopia II: Peri-Neural Canal Scleral Bowing and Choroidal Thickness in High Myopia—An American Ophthalmological Society Thesis | Association between Sight-Threatening Eye Diseases and Death by Suicide in South Korea A Nationwide Population-based Cohort Study |

© 2025 Rajeswaren V et al. *JAMA Network Open.*

| | | |
|---|---|---|
| Association of Risk Variants in the CFH Gene With Elevated Levels of Coagulation and Complement Factors in Idiopathic Multifocal Choroiditis | Differentiating Glaucomatous Optic Neuropathy From Non-glaucomatous Optic Neuropathies Using Deep Learning Algorithms | Open-Globe Injury Repairs in the American Academy of Ophthalmology IRIS® Registry 2014 – 2018 Incidence, Risk Factors, and Visual Outcomes |
| Prevalence of Diabetic Retinopathy in the US in 2021 | Posterior Staphyloma as Determining Factor for Myopic Maculopathy | Genetic Status Affects Disease-Specific Mortality But Not the Incidence of Local Recurrence in Patients with Uveal Melanoma |
| Low-Dose 0.01% Atropine Eye Drops vs Placebo for Myopia Control | Long-Term Endothelial Safety Profile With iStent Inject in Patients With Open-Angle Glaucoma | High Polygenic Risk Is Associated with Earlier Initiation and Escalation of Treatment in Early Primary Open-Angle Glaucoma |

© 2025 Rajeswaren V et al. *JAMA Network Open.*

| | | |
|---|---|---|
| Assessment of Ocular Deformation in Pathologic Myopia Using 3- Dimensional Magnetic Resonance Imaging | Efficacy of Adjuvants in Ophthalmic Regional Anesthesia: A Systematic Review and Network Meta-analysis | Epidemiologic Evaluation of Retinopathy of Prematurity Severity in a Large Telemedicine Program in India Using Artificial Intelligence |
| Evaluating Access to Laser Eye Surgery by Driving Times Using Medicare Data and Geographical Mapping | Exploring Imaging Characteristics Associated With Disease Activity in Idiopathic Multifocal Choroiditis: A Multimodal Imaging Approach | Management and Outcomes of Posterior Persistent Fetal Vasculature |
| Objectively Measured Visual Impairment and Dementia Prevalence in Older Adults in the US | Three-Dimensional Quantitative Description of the Implantable Collamer Lens in the Ocular Anterior Segment of Patients With Myopia | Global Trends in Blindness and Vision Impairment Resulting from Corneal Opacity 1984–2020<br>A Meta-analysis |

© 2025 Rajeswaren V et al. *JAMA Network Open.*

| | | |
|---|---|---|
| Association Between Genotype and Phenotype Severity in ABCA4-Associated Retinopathy | Endogenous Endophthalmitis From a Klebsiella pneumoniae Liver Abscess: The Incidence, Risk Factors, and Utility of Imaging | National Physician-Level Endophthalmitis Rates for Cataract Surgery among Medicare Beneficiaries in the United States: 2011–2019 |
| Effect of High-Dose Intravitreal Aflibercept, 8 mg, in Patients With Neovascular Age-Related Macular Degeneration: The Phase 2 CANDELA Randomized Clinical Trial | Inter-observer Variability of Clinical Activity Score: Assessments in Patients With Thyroid Eye Disease | Mitigating Hydroxychloroquine Toxicity with a Clinical Decision Support Tool |
| Use and Cost of Sustained- Release Corticosteroids for Cataract Surgery Under the Medicare Pass- | Benefits of a Laser Chorioretinal Anastomosis Plus Ranibizumab vs Ranibizumab Alone for Central Retinal Vein Occlusion: 4-Year | Association of Systemic Medication Use with Glaucoma and Intraocular Pressure The European Eye Epidemiology Consortium |

© 2025 Rajeswaren V et al. *JAMA Network Open.*

| | | |
|---|---|---|
| Through Program | Results | |

© 2025 Rajeswaren V et al. *JAMA Network Open.*

| | | |
|---|---|---|
| Refractive Accuracy and Visual Outcome by Self- Refraction Using Adjustable-Focus Spectacles in Young Children: A Randomized Clinical Trial | Increased Risks of Open-Angle Glaucoma in Untreated Hypertension | Causes of Childhood Blindness in the United States Using the IRIS® Registry (Intelligent Research in Sight) |
| Assessing Strategies to Reduce the Carbon Footprint of the Annual Meeting of the American Academy of Ophthalmology | Improving Effective Lens Position Prediction for Transscleral Fixation of Intraocular Lens Among Congenital Ectopia Lentis Patients | Intravitreal Therapy for Uveitic Macular Edema—Ranibizumab versus Methotrexate versus the Dexamethasone Implant<br>The MERIT Trial Results |
| GJA3 Genetic Variation and Autosomal Dominant Congenital Cataracts and Glaucoma Following Cataract Surgery | Peripheral Retinal Nonperfusion in Pediatric Patients with Optic Disc Hypoplasia | Immediate Sequential Bilateral Surgery in Refractive Lens Exchange Patients Clinical Outcomes and Adverse Events |

© 2025 Rajeswaren V et al. *JAMA Network Open.*

| | | |
|---|---|---|
| Three-Dimensional Structural Phenotype of the Optic Nerve Head as a Function of Glaucoma Severity | Evaluation of Dry Eye With Videokeratographer Using a Newly Developed Indicator | One-Year Brolucizumab Outcomes in Neovascular Age-Related Macular Degeneration from a Large United States Cohort in the IRIS® Registry |
| Brief Report: Role of Sex in Participation During Virtual Grand Rounds in Ophthalmology | Retinopathy of Prematurity Outcomes of Neonates Meeting Only a Single Screening Criterion: Proposal of the TWO- ROP Algorithm | Surgery, Tissue Plasminogen Activator, Antiangiogenic Agents, and Age-Related Macular Degeneration Study<br>A Randomized Controlled Trial for Submacular Hemorrhage Secondary to Age-Related Macular Degeneration |
| Association Between Health Insurance and Primary Care Vision Testing Among Children and Adolescents | Efficacy of Repeated Low-Level Red-Light Therapy for Slowing the Progression of Childhood Myopia: A Systematic Review and Meta-analysis | Side Effects of Proton Beam Radiotherapy Treatment on Iris Melanoma |

© 2025 Rajeswaren V et al. *JAMA Network Open.*

| | | |
|---|---|---|
| Ocular Symptoms in Adolescents and Young Adults with Electronic Cigarette, Cigarette, and Dual Use | Incidence and Characteristics of Facial and Ophthalmic Injuries From Domestic Mammal Bites | Ophthalmologist Turnover in the United States<br>Analysis of Workforce Changes from 2014 through 2021 |
| Sex and Racial and Ethnic Diversity Among Ophthalmology Subspecialty Fellowship Applicants | Ten-Year Incidence of Fibrosis and Risk Factors for Its Development in Neovascular Age-Related Macular Degeneration | Trends in Ophthalmology Practice Consolidation<br>2015–2022 |
| Calcium Channel Blocker Use and Associated Glaucoma and Related Traits Among UK Biobank Participants | Detection of Keratoconus With a New Corvis Biomechanical Index Optimized for Chinese Populations | Predictors of Poor Visual Outcome in Myelin Oligodendrocyte Glycoprotein-Related Optic Neuritis |

© 2025 Rajeswaren V et al. *JAMA Network Open.*

| | | |
|---|---|---|
| Rapid Point-of-Care Identification of Aspergillus Species in Microbial Keratitis | Pathogenic Variants in CEP290 or IQCB1 Cause Earlier-Onset Retinopathy in Senior-Loken Syndrome Compared to Those in INVS, NPHP3, or NPHP4 | Follow-up Extension Up to 43 Years of Modified Osteo-Odonto-Keratoprosthesis |
| Gender Bias and Ophthalmology Accreditation Council for Graduate Medical Education Milestones Evaluations | Short-Term Outcomes of Modified Boston Type-II Keratoprosthesis Implantation With Autologous Auricular Cartilage Reinforcement | Cystoid Macular Edema after Cataract Surgery in the United States IRIS® Registry (Intelligent Research in Sight) Analysis |
| Efficacy and Safety of 0.01% and 0.02% Atropine for the Treatment of Pediatric Myopia Progression Over 3 Years A Randomized Clinical Trial | EE-Explorer: A Multimodal Artificial Intelligence System for Eye Emergency Triage and Primary Diagnosis | Lotilaner Ophthalmic Solution 0.25% for Demodex Blepharitis Randomized, Vehicle-Controlled, Multicenter, Phase 3 Trial (Saturn-2) |

© 2025 Rajeswaren V et al. *JAMA Network Open.*

| | | |
|---|---|---|
| Ophthalmology Faculty Diversity Trends in the US | NOV03 for Signs and Symptoms of Dry Eye Disease Associated With Meibomian Gland Dysfunction: The Randomized Phase 3 MOJAVE Study | The Association of Physical Activity with Glaucoma and Related Traits in the UK Biobank |
| Diverse Research Teams and Underrepresented Groups in Clinical Studies | Electroretinographic Responses in Retinopathy of Prematurity Treated Using Intravitreal Bevacizumab or Laser | The Impact of Social Determinants of Health on Eye Care Utilization in a National Sample of People with Diabetes |
| Presumed Silicone Oil Droplets After Intravitreal Pegcetacoplan Injections | Long-term Impact of Immediate Versus Delayed Treatment of Early Glaucoma: Results From the Early Manifest Glaucoma Trial | Tumor-Associated Retinal Pigmentation in Choroidal Melanoma |

© 2025 Rajeswaren V et al. *JAMA Network Open.*

| | | |
|---|---|---|
| Vision Testing for Adolescents in the US | Progressive Visual Field Loss and Subsequent Quality of Life Outcomes in Glaucoma | Social Deprivation and the Risk of Screening Positive for Glaucoma in the MI-SIGHT Telemedicine-Based Glaucoma Detection Program |
| Outbreak of Bilateral Endophthalmitis After Immediate Sequential Bilateral Cataract Surgery | Gel Stent Versus Trabeculectomy: The Randomized, Multicenter, Gold- Standard Pathway Study (GPS) of Effectiveness and Safety at 12 Months | SCORE2 Report 24<br>Nonlinear Relationship of Retinal Thickness and Visual Acuity in Central Retinal and Hemiretinal Vein Occlusion |
| Bruton Tyrosine Kinase Inhibitor in 2 Patients With Vitreoretinal Lymphoma | Imaging Biomarkers of Mesopic and Dark-Adapted Macular Functions in Eyes With Treatment-Naïve Mild Diabetic Retinopathy | Retinal Nerve Fiber Layer Optical Texture Analysis<br>Detecting Axonal Fiber Bundle Defects in Patients with Ocular Hypertension |

© 2025 Rajeswaren V et al. *JAMA Network Open.*

| | | |
|---|---|---|
| Risk of Stroke, Myocardial Infarction, and Death After Retinal Artery Occlusion | 10-Year Clinical Outcomes of Tube Shunt Surgery at a Tertiary Care Center | Race, Ethnicity, Insurance, and Population Density Associations with Pediatric Strabismus and Strabismic Amblyopia in the IRIS® Registry |
| Neurodevelopmental Outcomes in Infants Screened for Retinopathy of Prematurity | Decreased Central Macular Choriocapillaris Perfusion Correlates With Increased Low Luminance Visual Acuity Deficit | Regional Disparities in Pediatric Uveitis Care Availability in the United States |
| Incidence and Risk Factors for Neutropenia After Intra- Arterial Chemotherapy for Retinoblastoma | Different patterns in the corneal endothelial cell loss after pars plana and pars limbal insertion of the Baerveldt glaucoma implan | United States Population Disparities in Ophthalmic Care<br>Blindness and Visual Impairment in the IRIS® Registry (Intelligent Research in Sight) |

© 2025 Rajeswaren V et al. *JAMA Network Open.*

| | | |
|---|---|---|
| Pathogen Surveillance for Acute Infectious Conjunctivitis | Gender Distribution and Trends of Ophthalmology Subspecialties, 1992-2020 | Evaluation of the Consistency of Glaucomatous Visual Field Defects Using a Clustered SITA-Faster Protocol |
| Psychiatric Comorbidities Associated With Keratoconus | Deep Learning–Based Estimation of Implantable Collamer Lens Vault Using Optical Coherence Tomography | Placebo Effect and Its Determinants in Ocular Hypotensive Therapy Meta-analysis and Multiple Meta-regression Analysis |
| Efficacy and Safety of Brolucizumab for Diabetic Macular EdemaThe KINGFISHER Randomized Clinical Trial | Endophthalmitis Associated With XEN Stent Implantation | Long-term Outcomes in Patients Undergoing Surgery for Primary Congenital Glaucoma between 1991 and 2000 A Single-Center Database Study |

© 2025 Rajeswaren V et al. *JAMA Network Open.*

| | | |
|---|---|---|
| Social Determinants of Health and Perceived Barriers to Care in Diabetic Retinopathy Screening | Optical Coherence Tomography Angiography Assessment in Congenital Aniridia | Prevalence and Associations of Nonglaucomatous Optic Nerve Atrophy in High Myopia<br>The Ural Eye and Medical Study |
| Intraocular Pressure in Eyes With Retinal Vein Occlusion Compared With Fellow Eyes Study of Comparative Treatments for Retinal Vein Occlusion 2 (SCORE2)<br>Report 27 | Risks of Topical Carbonic Anhydrase Inhibitors in Glaucoma Patients With Chronic Kidney Disease: A Nationwide Population-Based Study | Prognostication in Stargardt Disease Using Fundus Autofluorescence: Improving Patient Care |
| Rising Incidence of Syphilitic Uveitis–Related Hospitalizations | A Simplified Method to Minimize Systematic Bias of Single- Optimized Intraocular Lens Power Calculation | The Occurrence of Intraretinal and Subretinal Fluid in Anterior Ischemic Optic Neuropathy<br>Pathogenesis, Prognosis, and Treatment |

© 2025 Rajeswaren V et al. *JAMA Network Open.*

| | | |
|---|---|---|
| in the US | Formulas | |

© 2025 Rajeswaren V et al. *JAMA Network Open.*

| | | |
|---|---|---|
| Topical Atropine for Childhood Myopia ControlThe Atropine Treatment Long-Term Assessment Study | Thyroid Eye Disease and its Vision-Threatening Manifestations in the Academy IRIS Registry: 2014-2018 | Use of Mitomycin C in Dacryocystorhinostomy<br>A Report by the American Academy of Ophthalmology |
| Global, Regional, and National Epidemiology of Visual Impairment in Working-Age Individuals, 1990-2019 | A Photodynamic Therapy Index for Central Serous Chorioretinopathy to Predict Visual Prognosis Using Pretreatment Factors | Levodopa/Carbidopa to Augment the Treatment of Amblyopia<br>A Report by the American Academy of Ophthalmology |
| Local Firework Restrictions and Ocular Trauma | Optic Nerve Head Capillary Network Quantified by Optical Coherence Tomography Angiography and Decline of Renal Function in Type 2 Diabetes: A Three-Year Prospective Study | Royal College of Ophthalmologists' National Ophthalmology Database, Report 10: Risk Factors for Post–Cataract Surgery Endophthalmitis |

© 2025 Rajeswaren V et al. *JAMA Network Open.*

| | | |
|---|---|---|
| Surgical Approach and Reoperation Risk in Intermittent Exotropia in the IRIS Registry | Factors Affecting Visual Acuity and Central Visual Function in Glaucoma Patients With Myopia | Ophthalmology Database, Report 10: Risk Factors for Post–Cataract Surgery Endophthalmitis |
| Metformin Use and Age-Related Macular Degeneration in Patients Without Diabetes | Ocular Phenotypes in Patients With Hemophagocytic Lymphohistiocytosis: A Retrospective Analysis in a Single Center Over 7 Years | Trends in the Diagnosed Prevalence and Incidence of Major Eye Diseases in Medicare Part B Fee-for-Service Beneficiaries 68 Years of Age or Older |
| Interventions for Dry Eye An Overview of Systematic Reviews | Topical Betamethasone Treatment of Stevens-Johnson Syndrome and Toxic Epidermal Necrolysis with Ocular Involvement in the Acute Phase | Descemet Stripping Automated Endothelial Keratoplasty versus Descemet Membrane Endothelial Keratoplasty for Fuchs Endothelial Corneal Dystrophy: A National Registry-Based Comparison |

© 2025 Rajeswaren V et al. *JAMA Network Open.*

| | | |
|---|---|---|
| Axial Elongation Trajectories in Chinese Children and Adults With High Myopia | Vision Outcomes of Long-Term Immunomodulatory and Steroid Therapy in Sympathetic Ophthalmia | Use of Immunosuppression and the Risk of Subsequent Overall or Cancer Mortality |
| Risk of Falls and Fractures  in Individuals With Cataract, Age-Related Macular Degeneration, or Glaucoma | Myopia Control Efficacy of Spectacle Lenses With Aspherical Lenslets: Results of a 3-Year Follow-Up Study | Risk of Noninfectious Uveitis after Coronavirus Disease 2019 Vaccination in a United States Claims Database |
| Alzheimer Disease Treatment With Acetylcholinesterase Inhibitors and Incident Age- Related Macular Degeneration | Comparing Rates of Change in Moderate to Advanced Glaucoma: Retinal Nerve Fiber Layer Versus Bruch Membrane Opening-Minimum Rim Width | High Myopia Normative Database of Peripapillary Retinal Nerve Fiber Layer Thickness to Detect Myopic Glaucoma in a Chinese Population |

© 2025 Rajeswaren V et al. *JAMA Network Open.*

| | | |
|---|---|---|
| Green Space Morphology and School Myopia in China | Safety and Efficacy of Twice-Daily Pilocarpine HCl in Presbyopia: The Virgo Phase 3, Randomized, Double-Masked, Controlled Study | Detection of Circulating Tumor Cells in Patients with Small Choroidal Melanocytic Lesions |
| Sex or Gender Reporting in Ophthalmology Clinical Trials Among US Food and Drug Administration Approvals, 1995 to 2022 | Race Distribution in Nonarteritic Anterior Ischemic Optic Neuropathy | Neuro-ophthalmology Emergency Department and Inpatient Consultations at a Large Academic Referral Center |
| Intra-Anterior Chamber Injection of Ranibizumab in Advanced Pediatric Vitreoretinal Diseases | Intraocular Lens Power Calculations in Keratoconus Eyes Comparing Keratometry, Total Keratometry, and Newer Formulae | Zellweger Spectrum Disorder Ophthalmic Findings from a New Natural History Study Cohort and Scoping Literature Review |

© 2025 Rajeswaren V et al. *JAMA Network Open.*

| | | |
|---|---|---|
| Epidermal Growth Factor Receptor Inhibitors for Lung Cancer and the Risk of Keratitis | Prevalence of Visual Impairment and Availability of Eye Care Providers in Florida | Coats-like Vasculopathy in Inherited Retinal Disease<br>Prevalence, Characteristics, Genetics, and Management |
| Sex Disparities in Ophthalmology From Training Through Practice<br>A Systematic Review | Natural History of Visual Dysfunction in ABCA4 Retinopathy and Its Genetic Correlates | Thermal Pulsation in the Management of Meibomian Gland Dysfunction and Dry Eye<br>A Report by the American Academy of Ophthalmology |
| Optical Coherence Tomography Feature of Retinoschisis in CRB1-Associated Maculopathy | Multimodal Imaging Characteristics and Risk Factors Analysis of Waldenström Macroglobulinemia Retinopathy | Genome-Wide Meta-analysis Identifies Risk Loci and Improves Disease Prediction of Age-Related Macular Degeneration |

© 2025 Rajeswaren V et al. *JAMA Network Open.*

| | | |
|---|---|---|
| Four-Year Progression of Myopic Maculopathy in Children and Adolescents With High Myopia | First-in-Human Gene Therapy Trial of AAV8-hCARp.hCNGB3 in Adults and Children With CNGB3-associated Achromatopsia | Frequency and Patterns of Hearing Dysfunction in Patients Treated with Teprotumumab |
| Amblyopia Care Trends Following Widespread Photoscreener Adoption | Binocular Visual Outcomes Comparison of Two Trifocal Intraocular Lenses in High-Myopic Cataract Patients: A 1-Year Multicenter Study | Selective Laser Trabeculoplasty for the Treatment of Glaucoma<br>A Report by the American Academy of Ophthalmology |
| Diabetic Retinopathy and Quality of Life<br>A Systematic Review and Meta-Analysis | Onset and Progression of Persistent Choroidal Hypertransmission Defects in Intermediate Age-Related Macular Degeneration: A Novel Clinical Trial Endpoint | Efficacy of Different Powers of Low-Level Red Light in Children for Myopia Control |

© 2025 Rajeswaren V et al. *JAMA Network Open.*

| | | |
|---|---|---|
| Visual Impairment and Real- World Home Physical Activity With Home Environment in an Older Population | Analysis of Corneal and Lens Densitometry Changes in Patients With Type 1 Diabetes Mellitus | Prevalence and Cause of Loss of Visual Acuity and Visual Field in Highly Myopic Eyes<br>The Beijing Eye Study |
| Long-Term Risk and Prediction of Progression in Primary Angle Closure Suspect | Is Demodex Blepharitis Connected With Cataract Surgery? | Surgical Management of Full-Thickness Macular Holes in Macular Telangiectasia Type 2<br>A Global Multicenter Study |
| Sociodemographic and Clinical Predictors of Prolonged Length of Corneal Ulcer Hospitalizations | Most Common Ophthalmic Diagnoses in Eye Emergency Departments: A Multicenter Study | Optic Neuritis and Cranial Neuropathies Diagnosis Rates before Coronavirus Disease 2019, in the Initial Pandemic Phase, and Post-Vaccine Introduction |

© 2025 Rajeswaren V et al. *JAMA Network Open.*

| | | |
|---|---|---|
| Presence of Copy Number Variants Associated With Esotropia in Patients With Exotropia | The Impact of Social Determinants of Health on Vision Loss From Cataracts and Cataract Surgery Utilization in the United States—A National Health Interview Survey Analysis | Study of Late-Onset Stargardt Type 1 Disease<br>Characteristics, Genetics, and Progression |
| Risk of Herpes Zoster Ophthalmicus Recurrence After Recombinant Zoster Vaccination | A Review of the Prevalence of Ophthalmologic Diseases in Native American Populations | Eight-Year Outcomes of Bilateral Lateral Rectus Recessions versus Unilateral Recession-Resection in Childhood Basic-Type Intermittent Exotropia |
| Circulating Tumor DNA Posttreatment Measurements and Clinical Correlates in Retinoblastoma | The Role of the Retinal Nerve Fiber Layer Thickness on OCT in the Evaluation of Papillitis in Childhood Uveitis | Advanced Corneal Imaging in Keratoconus<br>A Report by the American Academy of Ophthalmology |

© 2025 Rajeswaren V et al. *JAMA Network Open.*

| | | |
|---|---|---|
| Gaps in the Vision Screening Pathway for School-Aged US Children | Fluctuation of Intraocular Pressure and Vascular Factors Are Associated With the Development of Epiretinal Membrane in Glaucoma | Imaging Features Associated with Persistent Avascular Retina in Retinopathy of Prematurity |
| Vision Impairment and Psychosocial Function in US Adults | Association Between Torque Teno Virus and Systemic Immunodeficiency in Patients With Uveitis With a Suspected Infectious Etiology | The Routine Use of Nonabsorbable Sutures in Bilateral Horizontal Rectus Recession |
| Uncorrected Refractive Error in the African American Eye Disease Study | Comprehensive Genotyping and Phenotyping Analysis of GUCY2D-Associated Rod- and Cone-Dominated Dystrophies | Ophthalmology Workforce Projections in the United States, 2020 to 2035 |

© 2025 Rajeswaren V et al. *JAMA Network Open.*

| | | |
|---|---|---|
| ATXN7-Related Cone-Rod DystrophyThe Integrated Functional Evaluation of the Cerebellum (CERMOI) Study | Scanning Laser Ophthalmoscopy Demonstrates Disc and Peripapillary Strain During Horizontal Eye Rotation in Adults | Relationship between Unstable Housing, Food Insecurity, and Vision Status in the MI-SIGHT Community Eye Disease Screening Program |
| Polarization-Sensitive OCT Imaging of Scleral Abnormalities in Eyes With High Myopia and Dome- Shaped Macula | Motion-Tracking Brillouin Microscopy Evaluation of Normal, Keratoconic, and Post– Laser Vision Correction Corneas | Early Endophthalmitis Incidence and Risk Factors after Glaucoma Surgery in the Medicare Population from 2016 to 2019 |
| Water-Free Cyclosporine Ophthalmic Solution vs Vehicle for Dry Eye DiseaseA Randomized Clinical Trial | Natural History of Optic Disc With Physiologic Large Cup: Incidence, Predictors of Glaucoma Conversion After Minimum 10-Year Follow-up | Impact of Vision Impairment and Ocular Morbidity and Their Treatment on Quality of Life in Children<br>A Systematic Review |

© 2025 Rajeswaren V et al. *JAMA Network Open.*

| | | |
|---|---|---|
| Phase 2 Trial Evaluating Minocycline for Geographic Atrophy in Age-Related Macular DegenerationA Nonrandomized Controlled Trial | Morning Blood Pressure Surge and Glaucomatous Visual Field Progression in Normal-Tension Glaucoma Patients With Systemic Hypertension | Critical Dependence on Area in Relationship between ARMS2/HTRA1 Genotype and Faster Geographic Atrophy Enlargement<br>Age-Related Eye Disease Study 2 Report Number 33 |
| Severe Intraocular Inflammation Following Intravitreal Faricimab | Efficacy of Vitrectomy With Tamponade Versus No Tamponade for Myopic Traction Maculopathy: A Multicenter Study (SCHISIS Report No.1) | Developing a Continuous Severity Scale for Macular Telangiectasia Type 2 Using Deep Learning and Implications for Disease Grading |
| Prevalence and Features of Fungal Keratitis Among US Patients With Commercial Health Insurance | One-Year Outcomes of Oral Treatment With Alga Capsules Containing Low Levels of 9-cis- β-Carotene in RDH5-Related Fundus Albipunctatus | Aqueous Shunts with Extraocular Reservoir for Open-Angle Adult Glaucoma<br>A Report by the American Academy of Ophthalmology |

© 2025 Rajeswaren V et al. *JAMA Network Open.*

| | | |
|---|---|---|
| Clinical Features and Treatment Outcomes of Carbapenem-Resistant Pseudomonas Aeruginosa Keratitis | Objective Quantification of Lens Opacity in Posterior Subcapsular Cataracts Using IOL Master 700 and CASIA-2 | Central Visual Field Testing in Early Glaucoma<br>A Report by the American Academy of Ophthalmology |
| Refractive Error Change and Overminus Lens Therapy for Childhood Intermittent Exotropia | Cataract Surgery in Patients With Uveitis Treated With Systemic Therapy in the Multicenter Uveitis Steroid Treatment (MUST) Trial and Follow-up Study: Risk Factors and Outcomes | Drug-Induced Uveitis Related to Checkpoint Inhibitors and MAP-kinase Inhibitors |
| Bias Reduction Practices in Underrepresented Groups in Ophthalmology Resident Recruitment | Incidence of and Risk Factors for Cataract in Anterior Uveitis | Diagnostic Odyssey of More than 1000 Patients with Inherited Retinal Diseases |

© 2025 Rajeswaren V et al. *JAMA Network Open.*

| | | |
|---|---|---|
| Genetic Associations of Primary Angle-Closure DiseaseA Systematic Review and Meta-Analysis | Lens-Sparing Surgery for Retrolental Stalk in Persistent Fetal Vasculature | Ab-Externo MicroShunt versus Trabeculectomy in Primary Open-Angle Glaucoma<br>Two-Year Results from a Randomized, Multicenter Study |
| Digital Technology Use Among Older Adults With Vision Impairment | A Systematic Review and Meta- analysis of Systemic Antihypertensive Medications With Intraocular Pressure and Glaucoma | The Orphan Drug for Acanthamoeba Keratitis (ODAK) Trial: PHMB 0.08% (Polihexanide) and Placebo versus PHMB 0.02% and Propamidine 0.1% |
| Access to Pediatric Eye Care by Practitioner Type, Geographic Distribution, and US Population Demographics | Using Objective Vision Measures to Explore the Association of Vision Impairment With Cognition Among Older Adults in the United States | Early-Onset Ocular Motor Cranial Neuropathy Is a Strong Predictor of Dementia: A Nationwide, Population-Based Cohort Study |

© 2025 Rajeswaren V et al. *JAMA Network Open.*

| | | |
|---|---|---|
| Representation of Women Among Individuals With Mild Variants in ABCA4-Associated Retinopathy<br>A Meta-Analysis | Association Between Sociodemographic Factors and Vision Difficulty in the National Health Interview Survey: A Population-Based Analysis | Risk of Alzheimer's Disease and Related Dementias in Persons with Glaucoma<br>A National Cohort Study |
| Mental Health Conditions Associated With Strabismus in a Diverse Cohort of US Adults | Progression of Pentosan Polysulfate Sodium Maculopathy in a Prospective Cohort | Subclinical Keratoconus Detection and Characterization Using Motion-Tracking Brillouin Microscopy |
| Patching in Children With Unilateral Congenital Cataract and Child Functioning and Parenting Stress | Four-Point Flange Intrascleral Fixation With Double Suture Through the Dislocated Plate-Haptic Trifocal Intraocular Lens | Retinal Artery and Vein Occlusion Risks after Coronavirus Disease 2019 or Coronavirus Disease 2019 Vaccination |

© 2025 Rajeswaren V et al. *JAMA Network Open.*

| | | |
|---|---|---|
| Home-Monitoring Vision Tests to Detect Active Neovascular Age-Related Macular Degeneration | Family History of Age-Related Macular Degeneration and Genetics Predict Progression to Advanced Age-Related Macular Degeneration Adjusting for Macular Status, Demographic, and Lifestyle Factors | Demographics, Practice Analysis, and Geographic Distribution of Neuro- Ophthalmologists in the United States in 2023 |
| COVID-19 Vaccine–Associated Uveitis in Patients With a History of Uveitis | Clinical Factors Associated With Long-Term OCT Variability in Glaucoma | Sensitivity, Specificity, and Cutoff Identifying Optic Atrophy by Macular Ganglion Cell Layer Volume in Syndromic Craniosynostosis |
| Rate of Initial Optic Nerve Head Capillary Density Loss and Risk of Visual Field Progression | Female Authorship and Ophthalmology Journal Editorial Board Membership Trends Over the Last Decade, 2012- 2021 | Visual Impairment from Uncorrected Refractive Error among Participants in a Novel Program to Improve Eye Care Access among Low-Income Adults in Michigan |

© 2025 Rajeswaren V et al. *JAMA Network Open.*

| | | |
|---|---|---|
| Place-Based Measures of Inequity and Vision Difficulty and Blindness | All Consecutive Ab Externo SIBS Microshunt Implantations With Mitomycin C: One-Year Outcomes and Risk Factors for Failure | Association of Private Equity Firm Acquisition of Ophthalmology Practices with Medicare Spending and Use of Ophthalmology Services |
| Pegcetacoplan Treatment and Consensus Features of Geographic Atrophy Over 24 Months | The Sensitivity of Ultra-Widefield Fundus Photography Versus Scleral Depressed Examination for Detection of Retinal Horseshoe Tears | Trabecular Procedures Combined with Cataract Surgery for Open-Angle Glaucoma<br>A Report by the American Academy of Ophthalmology |
| Daily Low-Level Red Light for Spherical Equivalent Error and Axial Length in Children With Myopia<br>A Randomized Clinical Trial | Corneal Collagen Cross-Linking for Progressive Keratoconus in Pediatric Patients: Up to 14 Years of Follow-up | Toric Monofocal Intraocular Lenses for the Correction of Astigmatism during Cataract Surgery: A Report by the American Academy of Ophthalmology |

© 2025 Rajeswaren V et al. *JAMA Network Open.*

| | | |
|---|---|---|
| Photokeratitis in Outdoor Event Participants Exposed to UV Radiation Display | Assessing Change in Exudative Age-Related Macular Degeneration With Macular Thickness Maps as a Surrogate Strategy for Remote Patient Monitoring | Visual Acuity Outcomes and Complications after Intraocular Lens Exchange<br>An IRIS® Registry (Intelligent Research in Sight) Analysis |
| Place, Race, and Lapses in Diabetic Retinopathy Care | Eosinophilic Angiocentric Fibrosis of the Orbit: A Clinicopathologic Review of 6 Novel Cases With Review of the Literature | Outcomes of Retinal Detachment after Open-Globe Injury, and Independent Validation of the Retinal Detachment after Open-Globe Injury Scoring System |
| Longitudinal Trends and Disparities in Diabetic Retinopathy Within an Aggregate Health Care Network | Outcomes of Intentionally Suspending Treatment in Eyes With Advanced Neovascular Age-Related Macular Degeneration | Elamipretide Topical Ophthalmic Solution for the Treatment of Subjects with Leber Hereditary Optic Neuropathy: A Randomized Trial |

© 2025 Rajeswaren V et al. *JAMA Network Open.*

| | | |
|---|---|---|
| Nationwide Prevalence of Inherited Retinal Diseases in the Israeli Population | Role of Static and Dynamic Ocular Biometrics Measured in the Dark and Light as Risk Factors for Angle Closure Progression | Ultra-Widefield–Guided Swept-Source OCT Findings of Peripheral Vitreoretinal Abnormality in Young Myopes |
| Re-Esterified Triglyceride ω-3 Fatty Acids in Dry Eye Disease With Meibomian Gland Dysfunction<br>A Randomized Clinical Trial | The Association of Vision Concerns With the Physical and Mental Well-being of Adolescents in the United States | Influence of a Capsular Tension Ring on Capsular Bag Behavior of a Plate Haptic Intraocular Lens: An Intraindividual Randomized Trial |
| Effect of Low-Dose Aspirin on the Course of Age- Related Macular Degeneration<br>A Secondary Analysis of the ASPREE | Risk Factors for Flat Anterior Chamber Requiring Intervention After Glaucoma Drainage Implant: A | Teprotumumab-Related Adverse Events in Thyroid Eye Disease: A |

© 2025 Rajeswaren V et al. *JAMA Network Open.*

| | | |
|---|---|---|
| Randomized Clinical Trial | Retrospective Case- Controlled Study | Multicenter Study |

© 2025 Rajeswaren V et al. *JAMA Network Open.*

| | | |
|---|---|---|
| Systemic Fluoroquinolone Use and Risk of Uveitis or Retinal Detachment | Microcatheter-Assisted Circumferential Trabeculotomy After Failed Glaucoma Surgeries in Childhood Glaucoma | Retinoblastoma in Asia: Clinical Presentation and Treatment Outcomes in 2112 Patients from 33 Countries |
| Melatonin and Risk of Age- Related Macular Degeneration | OCT Grading System of Macular Infarction Predicts Vision in Participants With Central Retinal or Hemiretinal Vein Occlusion: A Secondary Analysis of SCORE2 | Improved Staging of Ciliary Body and Choroidal Melanomas Based on Estimation of Tumor Volume and Competing Risk Analyses |
| Health Care Use for Eye Pain | Depression in Individuals With Diabetic Retinopathy in the US National Health and Nutrition Examination Survey, 2005-2008 | The Effect of Insurance Coverage Laws on Glaucoma Eyedrop Medication Usage |

© 2025 Rajeswaren V et al. *JAMA Network Open.*

| | | |
|---|---|---|
| Progression to Pars Plana Vitrectomy in Patients With Proliferative Diabetic Retinopathy | Development and Validation of the Low Vision Severely Constricted Peripheral Eyesight (LV-SCOPE) Questionnaire | ASCEND-Eye: Effects of Omega-3 Fatty Acids on Diabetic Retinopathy |
| Interventions for Proliferative Vitreoretinopathy | Clinical Outcome of Amniotic Membrane Transplant in Ocular Stevens–Johnson Syndrome/Toxic Epidermal Necrolysis at a Major Burn Unit | Disability-Adjusted Life Years Resulting from Ocular Injury among Deployed Service Members, 2001–2020 |
| Prophylactic Intracameral Antibiotics and Endophthalmitis After Cataract Surgery | Ocular Surface Evaluation in Immunoglobulin G4–Related Ophthalmic Disease | Evaluation of a Retinal Projection Laser Eyeware in Patients with Visual Impairment Caused by Corneal Diseases in a Randomized Trial |

© 2025 Rajeswaren V et al. *JAMA Network Open.*

| | | |
|---|---|---|
| Capsular Tension Ring Implantation for Intraocular Lens Decentration and Tilt in Highly Myopic Eyes: A Randomized Clinical Trial | Tube Versus Trabeculectomy IRIS Registry Study: 1-Year Secondary Treatment Outcomes | The Risk of Sympathetic Ophthalmia Associated with Open-Globe Injury Management Strategies A Meta-analysis |
| 0.01% Atropine Eye Drops in Children With Myopia and Intermittent ExotropiaThe AMIXT Randomized Clinical Trial | Ocular Manifestations and Outcomes in Children With Stevens–Johnson Syndrome and Toxic Epidermal Necrolysis: A Comparison With Adult Patients | Epidemiology of Pediatric Ocular Surface Inflammatory Diseases in the United States Using the Optum Labs Data Warehouse |
| Risk of Nonarteritic Anterior Ischemic Optic Neuropathy in Patients Prescribed Semaglutide | Influence of Goniotomy Size on Treatment Safety and Efficacy for Primary Open-Angle Glaucoma: A | Prevalence and Cost of Routine Preoperative Care for Low-Risk Cataract Surgery a Decade after Choosing Wisely |

© 2025 Rajeswaren V et al. *JAMA Network Open.*

| | | |
|---|---|---|
| | Multicenter Study | |

© 2025 Rajeswaren V et al. *JAMA Network Open.*

| | | |
|---|---|---|
| Gender, Race, and Ethnicity of US Academic Ophthalmology Faculty and Department Chairs From 1966 to 2021 | Comparison of Femtosecond Laser Assistance and Manual Trephination in Deep Anterior Lamellar Keratoplasty in the Treatment of Keratoconus: A Meta-Analysis | Bony Congenital Nasolacrimal Duct Obstruction<br>A Novel Phenotype of Aplasia of Lacrimal and Major Salivary Glands |
| Janus Kinase Inhibitor Therapy and Risk of Age- Related Macular Degeneration in Autoimmune Disease | A Novel Heterozygous TGFBI c. 1613C>A Pathogenic Variant is Associated With Lattice Corneal Dystrophy in a Chinese Family | The Efficacy and Safety of Standard versus Soft Topical Steroids after Cataract Surgery<br>A Systematic Review and Meta-analysis |
| Behavioral Risk Factor Surveillance System and American Community Survey Estimates of Vision | Parental Corneal Tomographic and Biomechanical Characteristics of Patients With Keratoconus | Association of Sociodemographic Characteristics with Pediatric Vision Screening and Eye Care: An Analysis of the 2021 National Survey of Children's Health |

© 2025 Rajeswaren V et al. *JAMA Network Open.*

| | | |
|---|---|---|
| Difficulty Prevalence | | |

© 2025 Rajeswaren V et al. *JAMA Network Open.*

| | | |
|---|---|---|
| Intraocular Pressure Outcomes After Lampalizumab Injections in Patients With Geographic Atrophy | Rotational Stability of Toric Capsular Bag–Fixated Intraocular Lenses in Duet Procedure for Reversible Trifocality | Use of Anterior Chamber Paracentesis for Diagnosis in Viral Anterior Uveitis |
| Antibiotic Treatment and Health Care Use in Children and Adolescents With Conjunctivitis | Characteristics Related to Visual Acuity Loss After Successful Photodynamic Therapy for Eyes With Central Serous Chorioretinopathy | Conbercept versus Laser for the Treatment of Infants with Zone II Retinopathy of Prematurity |
| | Minimum Corneal Diameter and Anterior Steep Axis Curvature Share the Same Meridian: A Novel Finding | Short-term Detection of Fast Progressors in Glaucoma<br>The Fast Progression Assessment through Clustered Evaluation (Fast-PACE) Study |

© 2025 Rajeswaren V et al. *JAMA Network Open.*

| | | |
|---|---|---|
| | Detailed Clinical, Ophthalmic, and Genetic Characterization of ADGRV1-Associated Usher Syndrome | Quantification and Predictors of Visual Field Variability in Healthy, Glaucoma Suspect, and Glaucomatous Eyes Using SITA-Faster |
| | Leber Hereditary Optic Neuropathy Gene Therapy: Longitudinal Relationships Among Visual Function and Anatomical Measures | Risk of Endophthalmitis Based on Cumulative Number of Anti-VEGF Intravitreal Injections |
| | Impact of Gender and Underrepresented in Medicine Status on Research Productivity Among Ophthalmology Residency Applicants | Deep Anterior Lamellar Keratoplasty Using Dehydrated versus Standard Organ Culture-Stored Donor Corneas Prospective Randomized Trial |

© 2025 Rajeswaren V et al. *JAMA Network Open.*

| | | |
|---|---|---|
| | Validation of a Published Model to Reduce Burden of Retinopathy of Prematurity Screening | Long-term Follow-up of a Phase 1/2a Clinical Trial of a Stem Cell-Derived Bioengineered Retinal Pigment Epithelium Implant for Geographic Atrophy |
| | Relationship of Choroidal Microvasculature Dropout and Beta Zone Parapapillary Area With Visual Field Changes in Glaucoma | Is Kidney Function Associated with Age-Related Macular Degeneration? Findings from the Asian Eye Epidemiology Consortium |
| | Topographic Correlation of Microperimetry With Structural Characteristics in Diabetic Macular Ischemia | Progressive Changes in the Neuroretinal Rim and Retinal Nerve Fiber Layer in Glaucoma Impact of Baseline Values and Floor Effects |

© 2025 Rajeswaren V et al. *JAMA Network Open.*

| | | |
|---|---|---|
| | Factors Associated With Incidental Retinal Emboli in the U.S. Adult Population | Faricimab Treat-and-Extend for Diabetic Macular Edema Two-Year Results from the Randomized Phase 3 YOSEMITE and RHINE Trials |
| | The Association Between Frailty and Visual Field Loss in US Adults | A Remote Consult Retinal Artery Occlusion Diagnostic Protocol |
| | Exudative Progression of Treatment-Naïve Nonexudative Macular Neovascularization in Age-Related Macular Degeneration: A Systematic Review With Meta-Analyses | Improved Outcomes with Heavy Silicone Oil in Complex Primary Retinal Detachment A Large Multicenter Matched Cohort Study |

© 2025 Rajeswaren V et al. *JAMA Network Open.*

| | | |
|---|---|---|
| | Progressive Changes in the Anterior Segment and Their Impact on the Anterior Chamber Angle in Primary Angle Closure Disease | Evaluating Primary Treatment for People with Advanced Glaucoma Five-Year Results of the Treatment of Advanced Glaucoma Study |
| | Alabama Screening and Intervention for Glaucoma and Eye Health through Telemedicine (AL-SIGHT): Baseline Results | ASCEND-Eye Effects of Aspirin on Diabetic Retinopathy |
| | Increased Risk of Optic Neuritis in Patients With Fibromyalgia: Nationwide Population-Based Cohort Study in South Korea | Intraocular Pressure Spike Following Stand-Alone Phacoemulsification in the IRIS® Registry (Intelligent Research in Sight) |

© 2025 Rajeswaren V et al. *JAMA Network Open.*

| | | |
|---|---|---|
| | Changes in Intraocular Lens Explantation Indications and Comparison of Various Explantation Techniques | Ophthalmic and Systemic Factors of Acute Nonarteritic Anterior Ischemic Optic Neuropathy in the Quark207 Treatment Trial |
| | Association of Bergmeister Papilla and Deep Optic Nerve Head Structures With Prelaminar Schisis of Normal and Glaucomatous Eyes | Hypotony Failure Criteria in Glaucoma Surgical Studies and Their Influence on Surgery Success |
| | Evaluating the Effect of a Myopia Control Spectacle Lens Among Children in Israel: 12- Month Results | Glycemic Trends in Patients with Thyroid Eye Disease Treated with Teprotumumab in 3 Clinical Trials |

© 2025 Rajeswaren V et al. *JAMA Network Open.*

| | | |
|---|---|---|
| | Risk of Stroke, Myocardial Infarction, Deep Vein Thrombosis, Pulmonary Embolism, and Death After Retinal Vein Occlusion | Association of Primary Open-Angle Glaucoma with Diabetic Retinopathy among Patients with Type 1 and Type 2 Diabetes<br>A Large Global Database Study |
| | Yield of Investigations in Young Patients Presenting With Transient Monocular Vision Loss: A Prospective Study | Incidence of Sympathetic Ophthalmia after Intraocular Surgery<br>A Systematic Review and Meta-analysis |
| | Antibiotics Versus Placebo for Acute Bacterial Conjunctivitis: Findings From a Cochrane Systematic Review | Post hoc Analysis of Role of Etanercept in Ocular Sequelae of Stevens–Johnson Syndrome/Toxic Epidermal Necrolysis |

© 2025 Rajeswaren V et al. *JAMA Network Open.*

| | | |
|---|---|---|
| | Salary Negotiations: Gender Differences in Attitudes, Priorities, and Behaviors of Ophthalmologists | Incident Noninfectious Uveitis Risk after Immune Checkpoint Inhibitor Treatment |
| | Striving Toward Better Eye Health Beyond Our Waiting Rooms: The LXXX Edward Jackson Memorial Lecture | Trends and Sociodemographic Patterns in Keratoconus Management 2015–2020<br>An American Academy of Ophthalmology IRIS® Registry Analysis |
| | Network Meta-analysis of Intraocular Lens Power Calculation Formula Accuracy in 1016 Eyes With Long Axial Length | Relationship between Intraocular Pressure Fluctuation and Visual Field Progression Rates in the United Kingdom Glaucoma Treatment Study |

© 2025 Rajeswaren V et al. *JAMA Network Open.*

| | | |
|---|---|---|
| | Comorbidity of Ocular and Facial Demodicosis | TENAYA and LUCERNE<br>Two-Year Results from the Phase 3 Neovascular Age-Related Macular Degeneration Trials of Faricimab with Treat-and-Extend Dosing in Year 2 |
| | Characterization of Ocular Injuries Caused by Orbeez Hydrated Gel Pellet Projectiles: Clinical Insights and Implications | Effectiveness of Conventional Digital Fundus Photography-Based Teleretinal Screening for Diabetic Retinopathy and Diabetic Macular Edema<br>A Report by the American Academy of Ophthalmology |
| | Tropicamide Versus Cyclopentolate for Cycloplegic Refraction in Pediatric Patients With Brown Irides: A Randomized Clinical Trial | Hospitalization, Overdose, and Mortality After Opioid Prescriptions Tied to Ophthalmic Surgery |

© 2025 Rajeswaren V et al. *JAMA Network Open.*

| | | |
|---|---|---|
| | Association Between Vision Difficulty and Sociodemographic Factors in Children: A Population-Based Analysis | Efficacy and Safety of Faricimab for Macular Edema due to Retinal Vein Occlusion 24-Week Results from the BALATON and COMINO Trials |
| | Infectious and Noninfectious Corneal Ulcers in Ocular Graft-Versus-Host Disease: Epidemiology, Clinical Characteristics, and Outcomes | Association of Cutaneous Keloids, Hypertrophic Scarring, and Fibrosis with Risk of Postoperative Proliferative Vitreoretinopathy |
| | Drusen and Other Retinal Findings in People With IgA Glomerulonephritis | Risk Factors for Meeting Criteria for Switching from Bevacizumab to Aflibercept When Treating Eyes with Diabetic Macular Edema and Visual Acuity of < 20/40 |

© 2025 Rajeswaren V et al. *JAMA Network Open.*

| | | |
|---|---|---|
| | Disparities in Promotion and Retention Rates Among Underrepresented in Medicine Faculty in U.S. Ophthalmology Departments | Cataract Surgery and Cognitive Benefits in the Older Person<br>A Systematic Review and Meta-analysis |
| | Magnetic Resonance Imaging of Globe Translation in Abducens Palsy | A Retrospective Longitudinal Study of 460 Patients with ABCA4-Associated Retinal Disease |
| | United States Regulatory Approval of Topical Treatments for Dry Eye | |

© 2025 Rajeswaren V et al. *JAMA Network Open.*

| | | |
|---|---|---|
| | Noninfectious Uveitis Risk After COVID-19 Vaccination: A Nationwide Retrospective Cohort Study | |
| | IMPG2 -Related Maculopathy | |
| | Development and Validation of a Novel Mobility Test for Rod-Cone Dystrophies: From Reality to Virtual Reality | |

© 2025 Rajeswaren V et al. *JAMA Network Open.*

| | | |
|---|---|---|
| | Optical Coherence Tomographic Optic Nerve Head Morphology in Myopia III: The Exposed Neural Canal Region in Healthy Eyes—Implications for High Myopia | |
| | Prevalence and Associations of Peripheral Arterial Disease in China: The Beijing Eye Study | |
| | The Humira in Ocular Inflammations Taper (HOT) Study | |

© 2025 Rajeswaren V et al. *JAMA Network Open.*

| | | |
|---|---|---|
| | Genetic and Environmental Contributions of Primary Angle- Closure Glaucoma and Primary Open-Angle Glaucoma: A Nationwide Study in Taiwan | |
| | Preoperative Risk Factors for Proptosis Recurrence After Rehabilitative Orbital Decompression in Graves' Orbitopathy Patients | |
| | RBP3-Retinopathy—Inherited High Myopia and Retinal Dystrophy: Genetic Characterization, Natural History, and Deep Phenotyping | |

© 2025 Rajeswaren V et al. *JAMA Network Open.*

| | | |
|---|---|---|
| | Nonsurgical Consecutive Exotropia Following Childhood Esotropia: A Multicentered Study | |
| | Risk of Herpes Zoster Ophthalmicus After COVID-19 Vaccination in a Large US Health Care Claims Database | |
| | Outcomes of Filtering Surgery Versus Clear Lens Extraction in Young Patients With Angle-Closure Glaucoma | |

© 2025 Rajeswaren V et al. *JAMA Network Open.*

| | | |
|---|---|---|
| | Exudation in Patients With Neovascular Age-Related Macular Degeneration Treated With the Port Delivery System or Monthly Injections | |
| | Impacts of Chronic Kidney Disease on Retinal Neurodegeneration: A Cross-Cohort Analysis | |
| | Lisch Epithelial Corneal Dystrophy Is Caused by Heterozygous Loss-of-Function Variants in MCOLN1 | |

© 2025 Rajeswaren V et al. *JAMA Network Open.*

| | | |
|---|---|---|
| | Performance of Corvis ST Parameters Including Updated Stress-Strain Index in Differentiating Between Normal, Forme-Fruste, Subclinical, and Clinical Keratoconic Eyes | |
| | The Epidemiology and Risk Factors for the Progression of Sympathetic Ophthalmia in the United States: An IRIS Registry Analysis | |
| | Corneal Sensitivity Is Inversely Correlated With Severity of Diabetic Retinopathy in a Predominantly Underrepresented Population | |

© 2025 Rajeswaren V et al. *JAMA Network Open.*

| | | |
|---|---|---|
| | Protective Effect of Amblyopia on Age-Related Macular Degeneration | |
| | Racial Differences in Diagnostic Accuracy of Retinal Nerve Fiber Layer Thickness in Primary Open-Angle Glaucoma | |
| | Prevalence and Severity of Glaucoma in the California Medicare Population | |

© 2025 Rajeswaren V et al. *JAMA Network Open.*

| | | |
|---|---|---|
| | Trends in Myopia and High Myopia from 1966 to 2019 in Olmsted County, Minnesota | |
| | Magnetic Resonance Imaging Findings and Genetic Testing Results in Children With Congenital Corneal Opacities | |
| | Prevalence and Economic Burden of Keratoconus in the United States | |

© 2025 Rajeswaren V et al. *JAMA Network Open.*

| | | |
|---|---|---|
| | Accuracy of the PEARL-DGS Formula for Intraocular Lens Power Calculation in Post–Myopic Laser Refractive Corneal Surgery Eyes | |
| | Effects of Decentration of Implantable Collamer Lens V4c on Visual Quality With the OPD-Scan III Aberrometer | |
| | Sex Disparities in Operating Room use Among Cataract Surgeons: A 10-Year Retrospective Population-Based Analysis | |

© 2025 Rajeswaren V et al. *JAMA Network Open.*

| | | |
|---|---|---|
| | Progression of PROM1-Associated Retinal Degeneration as Determined by Spectral-Domain Optical Coherence Tomography Over a 24-Month Period | |
| | Risk Factors for Failure in Glaucoma Patients Undergoing Microshunt Implantation | |
| | Prevalence and Risk Factors of Blindness Among Primary Angle Closure Glaucoma Patients in the United States: An IRIS Registry Analysis | |

© 2025 Rajeswaren V et al. *JAMA Network Open.*

| | | |
|---|---|---|
| | Effectiveness of Propranolol in Preventing Severe Retinopathy of Prematurity: A Comprehensive Systematic Review and Meta-Analysis | |
| | Inventory of Ocular Pulse Amplitude Values in Healthy Subjects and Patients With Ophthalmologic Illnesses: Systematic Review and Meta-analysis | |
| | Gender Representation Among Ophthalmology Fellowship Directors in 2022 | |

© 2025 Rajeswaren V et al. *JAMA Network Open.*

| | | |
|---|---|---|
| | FL-41 Tint Reduces Activation of Neural Pathways of Photophobia in Patients with Chronic Ocular Pain | |
| | Corneal Biomechanical Properties to Predict Prognosis of Abnormal Tomographic Corneas: A Prospective Cohort Study | |
| | Month 60 Imaging Findings and Relationship to Treatment Outcomes Following Anti-VEGF Therapy for Macular Edema Due to Central or Hemi-Retinal Vein Occlusion | |

© 2025 Rajeswaren V et al. *JAMA Network Open.*

| | | |
|---|---|---|
| | NSAIDs and Corticosteroids for the Postoperative Management of Age-Related Cataract Surgery: A Systematic Review and Meta- analysis | |
| | Sex Differences and Discordance Between Symptoms and Signs of Dry Eye Disease | |
| | Significance of Social Determinants of Health in Tumor Presentation, Hospital Readmission, and Overall Survival in Ocular Oncology | |

© 2025 Rajeswaren V et al. *JAMA Network Open.*

| | | |
|---|---|---|
| | Outcomes and Complications 5 Years After Surgery for Pediatric Cataract Associated With Persistent Fetal Vasculature | |
| | Noninfectious Outcomes of Intravitreal Antibiotic Steroid Injection and Topical Nonsteroidal Antiinflammatory Drugs Versus Triple Drop Therapy After Cataract Surgery | |
| | Retrospective Analysis of Radiation-Induced Complications of Uveal Melanoma Patients Treated With Brachytherapy in the Era of Anti-VEGF | |

© 2025 Rajeswaren V et al. *JAMA Network Open.*

| | | |
|---|---|---|
| | Time to Glaucoma Progression Detection by Optical Coherence Tomography in Individuals of African and European Descents | |
| | KESTREL and KITE Phase 3 Studies: 100-Week Results With Brolucizumab in Patients With Diabetic Macular Edema | |
| | Primary Practice Emphasis Area and Diversity Among Board- Certified Ophthalmologists | |

© 2025 Rajeswaren V et al. *JAMA Network Open.*

| | | |
|---|---|---|
| | Retinoblastoma Outcomes in the Americas: A Prospective Analysis of 491 Children With Retinoblastoma From 23 American Countries | |
| | Differences Between Keratometry and Total Keratometry Measurements in a Large Dataset Obtained With a Modern Swept Source Optical Coherence Tomography Biometer | |
| | Comparison of Eye Tracking and Teller Acuity Cards for Visual Acuity Assessment in Pediatric Cortical/Cerebral Visual Impairment | |

© 2025 Rajeswaren V et al. *JAMA Network Open.*

| | | |
|---|---|---|
| | Two-Year Myopia Management Efficacy of Extended Depth of Focus Soft Contact Lenses (MYLO) in Caucasian Children | |
| | Association Between Physical Indicators and Myopia in American Adolescents: National Health and Nutrition Examination Survey 1999-2008 | |
| | Correlation of Strabismus Surgical Outcomes Graded by Goal- Determined Metric With Patient Satisfaction Survey | |

© 2025 Rajeswaren V et al. *JAMA Network Open.*

| | | |
|---|---|---|
| | Clinical Characteristics and Associated Factors to the Development of Glaucoma in Eyes With Myopic Optic Neuropathy | |
| | Two-Year Performance and Safety Results of the MINIject Supraciliary Implant in Patients With Primary Open-Angle Glaucoma: Meta-Analysis of the STAR-I, II, III Trials | |
| | Exploring Patient Demographics and Presence of Retinal Vascular Disease in Paracentral Acute Middle Maculopathy | |

© 2025 Rajeswaren V et al. *JAMA Network Open.*

| | | |
|---|---|---|
| | Extending Peripheral Retinal Vascularization in Retinopathy of Prematurity Through Regulation of VEGF Signaling | |
| | Functional Vision in Patients With Biallelic USH2A Variants | |
| | Association of Neighborhood Opportunity With Severity of Retinoblastoma at Presentation | |

© 2025 Rajeswaren V et al. *JAMA Network Open.*

| | | |
|---|---|---|
| | The Influence of Lens Position, Vault Prediction, and Posterior Cornea on Phakic Posterior Chamber Intraocular Lens Power | |
| | Pigment Epithelial Detachment and Leak Point Locations in Central Serous Chorioretinopathy | |
| | Contemporary Patterns and Underlying Causes of Vitrectomy in Pediatric and Adolescent Patients: A Nationwide, Population-Based Analysis | |

© 2025 Rajeswaren V et al. *JAMA Network Open.*

| | | |
|---|---|---|
| | Social Determinants of Dry Eye in the United States: A Systematic Review | |
| | Bowman Layer Onlay Grafting as a Minimally Invasive Treatment for the Most Challenging Cases in Keratoconus | |
| | Practice Patterns and Sociodemographic Disparities in the Clinical Care of Anatomical Narrow Angles in the United States | |
| | Longitudinal Characteristics of Choroidal Neovascular Membrane in Pediatric Patients | |

© 2025 Rajeswaren V et al. *JAMA Network Open.*

| | | |
|---|---|---|
| | Detecting Fast Progressors: Comparing a Bayesian Longitudinal Model to Linear Regression for Detecting Structural Changes in Glaucoma | |

© 2025 Rajeswaren V et al. *JAMA Network Open.*

| | | |
|---|---|---|
| | Intraindividual Comparison of an Enhanced Monofocal and an Aspheric Monofocal Intraocular Lens of the Same Platform | |
| | Scleral Thickness in Simple Versus Complex Central Serous Chorioretinopathy | |
| | RP2-Associated X-linked Retinopathy: Clinical Findings, Molecular Genetics, and Natural History in a Large Cohort of Female Carriers | |
| | Features Associated With Vision in Eyes With Subfoveal Fibrosis From Neovascular Age-Related Macular Degeneration | |
| | Long-Term Effect of Systemic Comorbidity on Glaucoma Medication Adherence | |
| | OCT Optic Nerve Head Morphology in Myopia IV: Neural Canal Scleral Flange Remodeling in Highly Myopic Eyes | |

© 2025 Rajeswaren V et al. *JAMA Network Open.*

| | | |
|---|---|---|
| | Topical Antiseptics in Minimizing Ocular Surface Bacterial Load Before Ophthalmic Surgery: A Randomized Controlled Trial | |

© 2025 Rajeswaren V et al. *JAMA Network Open.*

| | | |
|---|---|---|
| | Association of Contrast Sensitivity With Eye Disease and Vision- Related Quality of Life | |
| | En Face Optical Coherence Tomography Illustrates the Trizonal Distribution of Drusen and Subretinal Drusenoid Deposits in the Macula | |
| | Donor Corneal Endothelial Cell Maturity and Its Impact on Graft Survival in Glaucoma Patients Undergoing Corneal Transplantation | |
| | Association Between Endothelial Cell Density and Corneal Thickness in Medium, Short, and Long Eyes of Han Chinese Cataract Patients | |
| | A Novel Risk Stratification-Based Immunomodulatory Treatment Strategy for Vogt-Koyanagi- Harada Disease | |
| | Cataract Surgery Outcomes in Retinitis Pigmentosa A Comparative Clinical Database Study | |

© 2025 Rajeswaren V et al. *JAMA Network Open.*

| | | |
|---|---|---|
| | The Development of a Thick-Lens Post–Myopic Laser Vision Correction Intraocular Lens Calculation Formula | |

© 2025 Rajeswaren V et al. *JAMA Network Open.*

| | | |
|---|---|---|
| | Intraocular Lens Power Calculation in Eyes After Myopic Laser Refractive Surgery and Radial Keratotomy: Bayesian Network Meta-analysis | |
| | Immunomodulatory Treatment Versus Systemic Steroids in Inflammatory Choroidal Neovascularization Secondary to Idiopathic Multifocal Choroiditis | |
| | Genetic Characteristics and Clinical Manifestations of Foveal Hypoplasia in Familial Exudative Vitreoretinopathy | |
| | Systemic Arterial and Venous Thrombotic Events Associated With Anti–Vascular Endothelial Growth Factor Injections: A Meta- Analysis | |
| | Endogenous Fungal Endophthalmitis: A Single-Center Retrospective Study and Review of the Literature | |

© 2025 Rajeswaren V et al. *JAMA Network Open.*

| | | |
|---|---|---|
| | Accuracy of Toric Intraocular Lens Calculations Using Estimated Versus Measured Posterior Corneal Astigmatism | |

© 2025 Rajeswaren V et al. *JAMA Network Open.*

| | | |
|---|---|---|
| | AFG3L2 and ACO2-Linked Dominant Optic Atrophy: Genotype–Phenotype Characterization Compared to OPA1 Patients | |
| | Uveitis Risk After the First Dose of COVID-19 Vaccination Based on Uveitis History: Matched Cohort and Crossover Case Series Study | |
| | Clinical, Diagnostic, and Treatment Characteristics of Orbital Liposarcoma | |
| | Incidence of Strabismus Post– Plaque Brachytherapy in Patients With Uveal Melanoma | |
| | Fetal Growth Restriction Leads to an Enlarged Cup-to-Disc Ratio in Adults Born at Full Term | |
| | Correlation Between Anterior Chamber Angle Status and Limbal Stem Cell Deficiency in Primary Angle-Closure Glaucoma | |
| | Outcomes of Strabismus Surgery Following | |

© 2025 Rajeswaren V et al. *JAMA Network Open.*

| | | |
|---|---|---|
| | Teprotumumab Therapy | |
| | Binocular Home Treatment for Amblyopia: Gains Stable for One Year | |
| | Severe Spontaneous Tilt of Scleral- Fixated Intraocular Lenses | |

© 2025 Rajeswaren V et al. *JAMA Network Open.*

| | | |
|---|---|---|
| | The Impact of Achieving Target Intraocular Pressure on Glaucomatous Retinal Nerve Fiber Layer Thinning in a Treated Clinical Population | |
| | Prevalence and Incidence of Strabismus by Age Group in Japan: A Nationwide Population- Based Cohort Study | |
| | Macular Neural and Microvascular Alterations in Type 2 Diabetes Without Retinopathy: A SS-OCT Study | |
| | The LISA-PPV Formula: An Ensemble Artificial Intelligence- Based Thick Intraocular Lens Calculation Formula for Vitrectomized Eyes | |
| | Anti-myopia Spectacles: The Standard of Care in the Future? | |

© 2025 Rajeswaren V et al. *JAMA Network Open.*

| | | |
|---|---|---|
| | Aqueous Humor Liquid Biopsy as a Companion Diagnostic for Retinoblastoma: Implications for Diagnosis, Prognosis, and Therapeutic Options: Five Years of Progress | |
| | Genetics, Clinical Characteristics, and Natural History of PDE6B-Associated Retinal Dystrophy | |

© 2025 Rajeswaren V et al. *JAMA Network Open.*

| | | |
|---|---|---|
| | Omidenepag Isopropyl Versus Timolol in Patients With Glaucoma or Ocular Hypertension: Two Randomized Phase 3 Trials (SPECTRUM 4 and 3) | |
| | A Prospective, Observational, Non-interventional Clinical Study of Participants With Choroideremia: The NIGHT Study | |
| | Socioeconomic Disparities in Glaucoma Severity at Initial Diagnosis: A Nationwide Electronic Health Record Cohort Analysis | |
| | Ocular Neuromyotonia: Clinical Features, Diagnosis, and Outcomes | |
| | Ultra-Wide-Field Optical Coherence Tomography and Gaussian Curvature to Assess Macular and Paravascular Retinoschisis in High Myopia | |

© 2025 Rajeswaren V et al. *JAMA Network Open.*

| | | |
|---|---|---|
| | Association Between Sociodemographic Factors and Self-reported Glaucoma in the National Health Interview Survey: A Population-Based Analysis | |
| | Effect of a Patient Portal Reminder Message After No-Show on Appointment Reattendance in Ophthalmology: A Randomized Clinical Trial | |

© 2025 Rajeswaren V et al. *JAMA Network Open.*

| | | |
|---|---|---|
| | Effects of Deep Optic Nerve Head Structures on Bruch's Membrane Opening-Minimum Rim Width and Peripapillary Retinal Nerve Fiber Layer | |
| | Clinical Significance of Optic Disc Hemorrhage Size in Visual Field Progression in Glaucoma | |
| | Supine Positioning for Graft Attachment After Descemet Membrane Endothelial Keratoplasty: A Randomized Controlled Trial | |
| | Clinical Course and Visual Outcomes of Papilledema in Pediatric Cerebral Venous Sinus Thrombosis | |
| | Efficacy of Intravitreal Injections Anti-Vascular Endothelial Growth Factor Treatment for Radiation Retinopathy: A Systematic Review and Meta-analysis | |

© 2025 Rajeswaren V et al. *JAMA Network Open.*

| | | |
|---|---|---|
| | Risk of Corneal Graft Rejection and Vaccination: A Matched Case- Control Study From a United States Integrated Health Care System | |
| | Reactivation After Teprotumumab Treatment for Active Thyroid Eye Disease | |

© 2025 Rajeswaren V et al. *JAMA Network Open.*

| | | |
|---|---|---|
| | A Comparison of Chemodnervation to Incisional Surgery for Acute, Acquired, Comitant Esotropia: An International Study | |
| | Characteristics of Eyes With CRB1 -Associated EOSRD/LCA: Age- Related Changes | |
| | Genetic Variants of the Beta-Adrenergic Receptor Pathways as Both Risk and Protective Factors for Retinopathy of Prematurity | |
| | Early Diagnosis of Syndromic Congenital Cataracts in a Large Cohort of Congenital Cataracts | |
| | Diagnostic Accuracy of Artificial Intelligence-Based Automated Diabetic Retinopathy Screening in Real-World Settings: A Systematic Review and Meta-Analysis | |

© 2025 Rajeswaren V et al. *JAMA Network Open.*

| | | |
|---|---|---|
| | Diagnostic Utility of QuantiFERON-Gold Testing in Patients with Ocular Inflammation in a Low-Endemic Tuberculosis Region | |

© 2025 Rajeswaren V et al. *JAMA Network Open.*

| | | |
|---|---|---|
| | Efficacy and Safety of Lotilaner Ophthalmic Solution (0.25%) for the Treatment of Demodex Blepharitis: A GRADE Assessed Systematic Review and Meta-Analysis of Observational & Experimental Studies | |
| | Inner Choroidal Fibrosis: An Optical Coherence Tomography Biomarker of Severity in Chronic Central Serous Chorioretinopathy | |
| | Thyroid Eye Disease: Pilot Study Comparison Between Patients in United States-Based and India- Based Practices | |
| | Genetic and Clinical Features of ABCA4-Associated Retinopathy in a Japanese Nationwide Cohort | |
| | Comparison of Legacy and New No-History IOL Power Calculation Formulas in Postmyopic Laser Vision Correction Eyes | |

© 2025 Rajeswaren V et al. *JAMA Network Open.*

| | | |
|---|---|---|
| | A Randomized, Controlled Comparison of NCX 470, a Nitric Oxide-Donating Bimatoprost, and Latanoprost in Subjects with Open- Angle Glaucoma or Ocular Hypertension: The MONT BLANC Study | |

© 2025 Rajeswaren V et al. *JAMA Network Open.*

| | | |
|---|---|---|
| | Gonioscopy-Assisted Transluminal Trabeculotomy Outcomes Under Different Levels of Glaucoma Severity: A Multicenter, Comparative Study | |
| | Association of Ocular Manifestations of Marfan Syndrome With Cardiovascular Complications | |
| | Patterns and Disparities in Recorded Gonioscopy During Initial Glaucoma Evaluations in the United States | |
| | Effectiveness of the Spot tm Vision Screener With Variations in Ocular Pigments | |
| | Association of Long-Term Intraocular Pressure Variability and Rate of Ganglion Complex Thinning in Patients With Glaucoma | |
| | Peripheral Vision in Patients Following Intraocular Lens Implantation: A Systematic Review and Meta-Analysis | |

© 2025 Rajeswaren V et al. *JAMA Network Open.*

| | | |
|---|---|---|
| | En Face and Volumetric Comparison of Hypertransmission Defects Evaluated by Cirrus and Spectralis Optical Coherence Tomography | |

© 2025 Rajeswaren V et al. *JAMA Network Open.*

| | | |
|---|---|---|
| | Correlation of Refractive Error with Anisometropia Development in Early Childhood | |
| | Corneal Biomechanical Characteristics in Myopes and Emmetropes Measured by Corvis ST: A Meta-Analysis | |
| | External Validation of a Model to Predict Postoperative Globe Axial Length in Children After Bilateral Cataract Surgery | |
| | Association Between Quantitative and Qualitative Imaging Biomarkers and Geographic Atrophy Growth Rate | |
| | Eight Years and Beyond Longitudinal Changes of Peripapillary Structures on OCT in Adult Myopia | |
| | Factors Associated With Visual Field Testing Reliability in Children With Glaucoma or Suspected Glaucoma | |

© 2025 Rajeswaren V et al. *JAMA Network Open.*

| | | |
|---|---|---|
| | Racial Disparities in Glaucoma Vision Outcomes and Eye Care Utilization: An IRIS Registry Analysis | |

© 2025 Rajeswaren V et al. *JAMA Network Open.*

| | | |
|---|---|---|
| | IQCB1 (NPHP5)-Retinopathy: Clinical and Genetic Characterization and Natural History | |
| | Retinal Ischemic Perivascular Lesions Are Associated With Myocardial Infarction in Patients With Coronary Artery Disease | |
| 525 | | |

© 2025 Rajeswaren V et al. *JAMA Network Open.*
