# Supplementary material for: Adherence to Updated Race and Ethnicity Reporting Guidance in Ophthalmology Journals
Source: JAMA Netw Open. 2025 Sep 2;8(9):e2529778. doi: 10.1001/jamanetworkopen.2025.29778 (PMC12406061; doi:10.1001/jamanetworkopen.2025.29778)
Supplement: Supplement 2. — Data Sharing Statement [file jamanetwopen-e2529778-s002.pdf]

Data Sharing Statement

Rajeswaren. Adherence to Updated Race and Ethnicity Reporting Guidance in Ophthalmology Journals. *JAMA Netw Open*. Published September 02, 2025.
doi:10.1001/jamanetworkopen.2025.29778

Data
Data available: Yes
Data types: Data (not involving human participants)
How to access data: hw9955@wayne.edu
When available: With publication

Supporting Documents
Document types: None

Additional Information
Who can access the data: Researchers whose proposed use of the data has been approved
Types of analyses: Research
Mechanisms of data availability: After approval of a proposal
